# Major Threat to Malaria Control Programs by *Plasmodium falciparum* Lacking Histidine-Rich Protein 2, Eritrea

**DOI:** 10.3201/eid2403.171723

**Published:** 2018-03

**Authors:** Araia Berhane, Karen Anderson, Selam Mihreteab, Karryn Gresty, Eric Rogier, Salih Mohamed, Filmon Hagos, Ghirmay Embaye, Anderson Chinorumba, Assefash Zehaie, Simone Dowd, Norman C. Waters, Michelle L. Gatton, Venkatachalam Udhayakumar, Qin Cheng, Jane Cunningham

**Affiliations:** Ministry of Health, Asmara, Eritrea (A. Berhane, S. Mihreteab, S. Mohamed, F. Hagos, G. Embaye);; Australian Defence Force Malaria and Infectious Disease Institute, Brisbane, Queensland, Australia (K. Anderson, K. Gresty, S. Dowd, Q. Cheng);; QIMR–Berghofer Medical Research Institute, Brisbane (K. Anderson, K. Gresty, S. Dowd, Q. Cheng);; Centers for Disease Control and Prevention, Atlanta, Georgia, USA (E. Rogier, V. Udhayakumar);; World Health Organization, Geneva, Switzerland (A. Chinorumba, J. Cunningham);; World Health Organization, Asmara (A. Zehaie);; Walter Reed Army Institute of Research, Silver Spring, Maryland, USA (N.C. Waters);; Queensland University of Technology, Brisbane (M.L. Gatton)

**Keywords:** Plasmodium falciparum, parasites, malaria, histidine-rich protein 2 gene, HRP2, histidine-rich protein 3 gene, HRP3, malaria control programs, rapid diagnostic tests, RDTs, HRP2 deletion, genetic diversity, genetic relatedness, Eritrea

## Abstract

False-negative results for *Plasmodium falciparum* histidine-rich protein (HRP) 2–based rapid diagnostic tests (RDTs) are increasing in Eritrea. We investigated HRP gene 2/3 (*pfhrp2*/*pfhrp3*) status in 50 infected patients at 2 hospitals. We showed that 80.8% (21/26) of patients at Ghindae Hospital and 41.7% (10/24) at Massawa Hospital were infected with *pfhrp2*-negative parasites and 92.3% (24/26) of patients at Ghindae Hospital and 70.8% (17/24) at Massawa Hospital were infected with *pfhrp3*-negative parasites. Parasite densities between *pfhrp2*-positive and *pfhrp2*-negative patients were comparable. All *pfhrp2*-negative samples had no detectable HRP2/3 antigen and showed negative results for HRP2-based RDTs. *pfhrp2*-negative parasites were genetically less diverse and formed 2 clusters with no close relationships to parasites from Peru. These parasites probably emerged independently by selection in Eritrea. High prevalence of *pfhrp2*-negative parasites caused a high rate of false-negative results for RDTs. Determining prevalence of *pfhrp2*-negative parasites is urgently needed in neighboring countries to assist case management policies.

Eritrea, which is located in the Horn of Africa, has reduced malaria mortality and incidence rates extensively over the past decade (*1**,**2*). This reduction is attributed largely to integrated vector management, early diagnosis, and effective treatment implemented by the national malaria control program (NMCP). However, malaria remains a public health concern because there were ≈65,000 cases and 5 million persons living in malaria-prone areas in 2015 (*3*). Malaria is unstable in Eritrea, and seasonal and transmission patterns vary across 3 ecologic zones. *Plasmodium falciparum* infection accounts for ≈70% of confirmed malaria cases and *P. vivax* for the remaining ≈30% (*4*).

Microscopy remains the mainstay of malaria diagnosis at hospitals. Rapid diagnostic tests (RDTs) were introduced at the community level and primary health facilities in 2006, which paved the way for implementation of artemisinin-based combinations as first-line treatment in 2007. RDTs that detect *P. falciparum* and *P. vivax* simultaneously by targeting histidine-rich protein 2 (HRP2) and *P. vivax* plasmodium lactate dehydrogenase (pLDH), and met the World Health Organization (WHO)–recommended procurement criteria (*5*) were implemented in Eritrea with a quality assurance program that included training operators regularly and testing RDT lots before distribution.

In 2014, the NMCP received reports of false-negative RDT results in microscopically confirmed cases of *P. falciparum* and *P. vivax* malaria at several health facilities. The RDTs involved were from 10 lots that had passed testing at a WHO–Foundation for Innovative New Diagnostics lot-testing laboratory. To address this issue, the Ministry of Health (MOH) conducted exploratory investigations at 12 health facilities located in 4 regions of Eritrea and used different brands of RDTs. Results showed an overall false-negative result rate of 80% (41/50) for microscopically confirmed cases of *P. falciparum* malaria (*6*).

Deficiencies in RDT storage and operational issues were ruled out as causes of the false-negative results. Furthermore, samples of deployed RDTs were retrieved from the field and retested at a WHO–Foundation for Innovative New Diagnostics lot-testing laboratory against well-characterized reference samples. All RDTs passed testing, further suggesting that RDT quality issues did not cause false-negative results for *P. falciparum* (*6*). The cause of false-negative results for *P. vivax* was believed to be low parasite density. On the basis of these findings, the MOH recalled the RDTs and investigated *P. falciparum–*specific parasite factors, such as presence of *P. falciparum* lacking HRP2, as a primary cause.

HRP2-based RDTs target parasite HRP2, and some cross-react with HRP3 because of sequence similarities between the 2 proteins. *P. falciparum* lacking HRP2 and HRP3 caused by deletions of genes encoding these antigens (*pfhrp2* and *pfhrp3*) in clinical cases were first reported in the Amazon region of Peru (*7*) and subsequently in Colombia (*8*), Suriname (*9*), and Brazil and Bolivia (*10*) (prevalence 4%–41%). The high prevalence of *pfhrp2*-negative parasites in Peru correlated with poor performance of HRP2-based RDTs (*11*) and led WHO to recommend using non–HRP2-based RDTs for case management in affected areas.

Outside South America, sporadic and low prevalence of *pfhrp2*-negative parasites has been reported in India (*12*); along the China–Myanmar border (*13*); and in countries in Africa, including Mali (*14*), Senegal (*15*), and the Democratic Republic of the Congo (*16*). A high prevalence (22%–41%) of *pfhrp2*-negative parasites was reported in 2 areas of Ghana (*17*). However, *pfhrp2*-negative parasites have not been detected in East Africa.

Unlike South America and Asia, where *P. vivax* infections dominate, most countries in Africa have predominantly *P. falciparum* infections. Thus, many of these countries have adopted HRP2-based RDTs only for *P. falciparum.* These RDTs do not detect *pfhrp2*-negative parasites, which results in *P. falciparum* infections not being detected and treated. Use of only HRP2-based RDTs in areas where *pfhrp2*-negative parasites are present will lead to increases in disease burden and transmission (*18*). Therefore, emergence of *pfhrp2*-negative parasites poses a serious threat to malaria control programs in Africa where malaria disease burden is high and RDT is the preferred diagnostic test. We investigated *P. falciparum* lacking *pfhrp2* and *pfhrp3* in Eritrea to determine whether *pfhrp2/pfhrp3* deletions were a cause of false-negative results for HRP2-based RDTs and to describe origins of parasites.

## Methods

### Study Site

**Figure 1:**
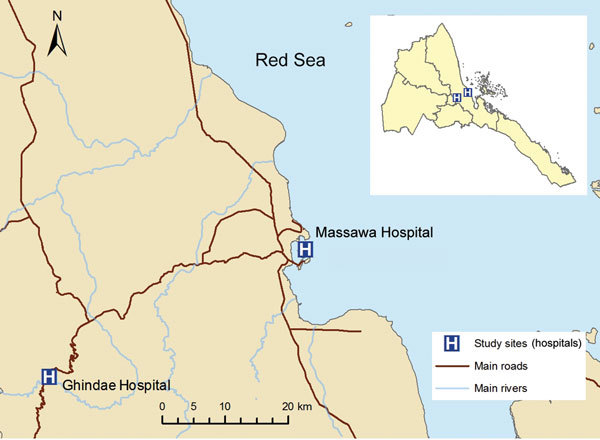
Location of study sites at Ghindae and Massawa Hospitals, Eritrea, for analysis of a major threat to malaria control programs by *Plasmodium falciparum* lacking histidine-rich protein 2. Inset shows the location of the study sites in Eritrea.

Eritrea is divided into 6 administrative regions. This study was conducted at Ghindae Hospital and Massawa Hospital in the Northern Red Sea Region (Figure 1) in March 2016. These locations were selected because of the timing of the malaria transmission season, easy access to infrastructure, and a high incidence of false-negative RDT results reported in this region (*6*).

### Patient Enrollment and Sample Collection

Consecutive nonpregnant persons >5 years of age who came to the 2 hospitals and confirmed as being infected with *P. falciparum* by microscopy and a pan-pLDH single test line RDT (Carestart Malaria pLDH(PAN) G0111; Access Bio, Inc., Somerset, NJ, USA) were invited to participate. Venous blood (50 µL) was obtained from each consenting patient, spotted onto Whatman (Brentford, UK) 3M filter paper, dried, stored in a zip-lock bag containing desiccant, and used to prepare thin and thick blood smears. Smears and dried blood spots were shipped to different laboratories for further analyses.

### Microscopy and HRP2-Based RDT

Blood smears were stained and examined at both hospitals and the Eritrean National Reference Laboratory (NRL; Asmara, Eritrea) according to the WHO microscopy manual (*19*). Final parasite species and density counts were determined by NRL microscopists who participated in the WHO/National Institute for Communicable Diseases malaria microscopy proficiency testing program. Blood samples were tested by using a combination HRP2/*P. falciparum* pLDH/*P. vivax* pLDH RDT (Bioline Malaria Ag Pf/Pf/Pv 05FK120; Standard Diagnostics, Gyeonhhi-do, South Korea) according to the manufacturer’s instructions.

### DNA Extraction and *Plasmodium* Speciation

Each dried blood spot was processed into 3 discs from which genomic DNA was extracted by using QIAamp DNA Mini Kits and a QIAcube Robot (QIAGEN, Crawley, UK) according to the manufacturer’s instructions. DNA was eluted into a volume of 100 µL, and 10 µL was used in each PCR. An 18S rRNA gene-based multiplex PCR (*20*) was used to determine whether 4 human *Plasmodium* spp. were present in each sample.

### Characterization of *pfhrp2* and *pfhrp3* Gene Deletions and Flanking Genes

Presence or absence of *pfhrp2* (Pf3D7_0831800) and *pfhrp3* (Pf3D7_1372200) genes was characterized by amplifying across exon1–exon2 and exon2 (*7*). Outcomes were classified as *pfhrp2*-positive or *pfhrp3*-positive (PCR result positive for exon1 and exon2 of the *pfhrp2* or *pfhrp3* gene) or *pfhrp2*-negative or *pfhrp3*-negative (PCR result negative for exon1 and exon2 of the *pfhrp2* or *pfhrp3* gene, but PCR positive result for 3 single-copy *P. falciparum–*specific genes [*P. falciparum* merozoite surface protein 1, *P. falciparum* merozoite surface protein 2, and *P. falciparum* glutmate-rich protein]) (*21*). Presence or absence of genes flanking *pfhrp2* (Pf3D7_0831900/MAL7P1_230 and Pf3D7_0831700/MAL7P1_228) and *pfhrp3* (Pf3D7_1372400/MAL12P1_485 and Pf3D7_1372100/MAL13P1_475) were characterized by PCR amplification of a fragment in each gene (*7*).

### HRP2 Levels

HRP2 levels were measured for each sample by using Luminex mulitplex bead-based immunoassay described elsewhere (*22*). Dried blood spots were incubated overnight in elution buffer (0.05% phosphate-buffered saline, Tween 20, 0.05% NaN_3_) to give a 1:20 dilution of whole blood. A 50-µL elution was used in each assay. The threshold mean fluorescence intensity minus background (MFI – bg) signal that indicated true negativity for HRP2 was derived by testing 86 blood samples from a setting to which malaria was not endemic. The MFI – bg positivity cutoff value was the lognormal mean ± 3 SD for this malaria-negative population.

### Microsatellite Analysis

We amplified 7 neutral microsatellite markers (TA1, PolyA, PfPK2, TA109, 2490, 313, and 383) from each sample (*23*) and assayed for size by using an ABI 3100 Genetic Analyzer (Applied Biosystems, Foster City, CA, USA). We scored alleles manually by using Peak Scanner Software version 1.0 (Applied Biosystems), using a height of 300 relative fluorescence units as the minimal peak threshold.

### Genetic Diversity and Population Genetic Analyses

We determined haplotypes for each parasite isolate from 7 microsatellite markers and used for genetic diversity and genetic relatedness analysis among parasites from Eritrea and between parasites from Eritrea and Peru (*23*) by using PHYLOViZ software (*24*). We calibrated sizes of 7 microsatellite markers against those of *P. falciparum* strain 3D7 before conducting genetic relatedness analysis, using a cutoff value of 2 (minimum differences for 2 loci) to compare parasites. We calculated expected heterozygosity (H*_E_*) values for each of the 7 microsatellite markers by using FSTAT software (https://www2.unil.ch/popgen/softwares/fstat.htm) and derived the mean H*_E_* for *pfhrp2*-positive and *pfhrp2*-negative parasite populations in Eritrea.

### Statistical Analysis

We conducted graphics and statistical analysis by using GraphPad Prism 7.00 for Windows (GraphPad Software, La Jolla, CA, USA). Parasite densities were log-transformed for analysis and the geometric mean is reported. We used a Mann-Whitney test to compare log parasite densities between hospitals and between *pfhrp2*-positive and *pfhrp2*-negative parasites.

### Ethics Considerations

The study was approved by the Eritrean MOH Research and Ethical Committees. The Northern Red Sea branch of the MOH and authorities of the 2 hospitals were informed, through an official letter, on the scope, coverage, and objectives of the study.

Patients were enrolled after providing consent following a detailed explanation about the investigation. Data from patients were recorded on structured forms. All patient specimens were given a unique identification number after collection, and only this number was used for data linkage.

Laboratory analyses of *pfhrp2/pfhrp3* status of parasites and their genetic diversity and relatedness was conducted at the Australian Defence Force Malaria and Infectious Disease Institute. Analyses were approved by the Australian Defence Joint Health Command Low Risk Ethics Panel (LREP 15–004).

## Results

### Patient Characteristics

A total of 51 patients recruited from the 2 hospitals participated in the study. We obtained characteristics for all of these patients (Table 1).

**Table 1:** Characteristics of patients infected with *Plasmodium falciparum* at 2 hospitals, Eritrea*

Characteristic	Ghindae	Massawa	Overall
No. enrolled	26	25	51
Mean age, y (range)	29.15 (10–60)	29.08 (7–68)	29.12 (7–68)
No. males:no. females (ratio)	16:10 (1.6:1)	13:12 (1.08:1)	29: 22 (1.32:1)
Travel history outside area of residence, no. positive/no. tested	2/26	3/25	5/51
Clinical history in previous 2 weeks, no. positive/no. tested			
Any malaria symptoms	25/26	24/25	49/51
Fever	10/26	24/25	34/51
Antimalarial treatment	0	0	0
Microscopy results			
No. *P. falciparum* positive	26	25	50
GM parasite density, parasites/μL (95% CI), [range]	6,851 (3,490–13,448) [16–71,000]	5,304 (2,620–10,734) [55–100,000]	6,059 (3,782–9,705) [16–100,000]
PCR results			
No. *P. falciparum* positive	26	24	50
No. *P. vivax* positive	0	1	1
RDT results			
No. pan-pLDH positive	26	25	51
No. HRP-based *P. falciparum* positive	5	14	19
False-negative result for *P. falciparum*, %	80.8	41.7	62.0

*GM, geometric mean; HRP2, histidine-rich protein 2; pLDH, *Plasmodium* lactate dehydrogenase; RDT, rapid diagnostic test.

### Microscopy and *Plasmodium* Speciation PCR

Microscopy at the NRL confirmed 50 infections only with *P*. *falciparum* and 1 infection only with *P. vivax*. These results were confirmed by PCR; no mixed infections or infections with other *Plasmodium* species were identified. The 1 infection with *P. vivax* was excluded from further analysis. The number of *P. falciparum–*infected patients was approximately equal between the hospitals, and patients from the 2 hospitals had comparable parasite densities (p = 0.47) (Table 1).

### RDT

All 50 confirmed *P. falciparum* samples showed positive results for the pan-pLDH RDT, but only 19 showed positive results for the HRP2-based RDT. These samples showed an overall false-negative rate of 62.0%; a total of 21 (80.8%) of 26 at Ghindae Hospital and 10 (41.7%) of 24 at Massawa Hospital were false negative (Table 1).

### Presence or Absence of *pfhrp2-* and *pfhrp3*-Negative Genes

**Figure 2:**
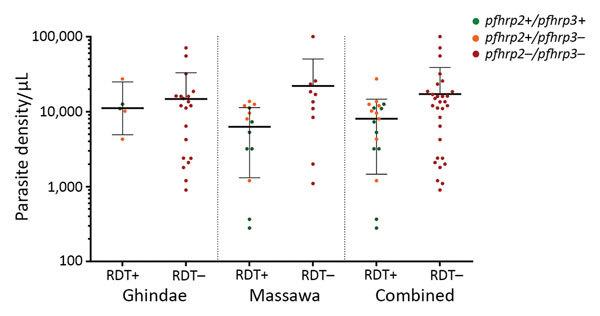
*Plasmodium falciparum* histidine-rich protein 2–based malaria RDT results and presence or absence of the *pfhrp2/pfhrp3* genes, in relation to parasite density, orizontal lines indicate geometric means a,d Eritrea. Horizontal lines indicate geometric means, and error bars indicate 95% CIs. *pfhrp, P. falciparum* histidine-rich protein; RDT, rapid diagnostic test; – negative; +, positive.

At Ghindae Hospital, 80.8% (21/26) of samples contained *pfhrp2*-negative parasites and 92.3% (24/26) *pfhrp3*-negative parasites. At Massawa Hospital, 41.7% (10/24) of samples contained *pfhrp2*-negative parasites and 70.8% (17/24) *pfhrp3*-negative parasites (Table 2). When we combined sample data, 62.0% (31/50) of samples contained *pfhrp2*-negative parasites and 82.0% (41/50) *pfhrp3*-negative parasites. All *pfhrp2*-negative samples (31/50, 62.0%) were also *pfhrp3* negative, but only 9 (18.0%) of 50 *pfhrp3*-negative samples were also *pfhrp2*-negative (Table 2; Figure 2).

**Table 2:** Frequency of *pfhrp2*, *pfhrp3*, and flanking genes in patients infected with *Plasmodium falciparum* at 2 hospitals, Eritrea*

Hospital	Upstream	*pfhrp2*	Downstream	No. (%)	Upstream	*pfhrp3*	Downstream	No. (%)
Ghindae	+	+	+	4 (15.4)	+	+	+	2 (7.7)
					+	–	–	2 (7.7)
	–	+	+	1 (3.9)	–	–	–	1 (3.9)
	–	–	+	21 (80.8)	–	–	–	21 (80.8)
Subtotal				26				26
Massawa	+	+	+	7 (29.2)	+	+	+	6 (25.0)
					+	–	–	1 (4.2)
	–	+	+	7 (29.2)	+	+	+	1 (4.2)
					+	–	–	6 (25.0)
	–	–	+	10 (41.7)	–	–	+	2 (8.3)
					–	–	–	8 (33.3)
Subtotal				24				24
Combined	+	+	+	11 (22.0)	+	+	+	8 (16.0)
					+	–	–	3 (6.0)
	–	+	+	8 (16.0)	+	+	+	1 (2.0)
					+	–	–	6 (12.0)
					–	–	–	1 (2.0)
	–	–	+	31 (62.0)	–	–	+	2 (4.0)
					–	–	–	29 (58.0)
Total				50				50

*Parasites with same presence or absence pattern for *pfhrp2* and flanking genes but different presence or absence pattern for *pfhrp3* and flanking genes are separated into subgroups. *pfhrp, P. falciparum* histidine-rich protein +, positive; –, negative.

### Presence or Absence of Genes Flanking *pfhrp2* and *pfhrp3*

All 31 *pfhrp2*-negative isolates had a deletion of the upstream gene but retained the downstream gene of *pfhrp2*. A total of 8 *pfhrp2*-positive samples had a deletion of the upstream gene. In contrast, of 41 *pfhrp3*-negative samples, 30 had deletions of both flanking genes, 2 had deletions of only the upstream gene, and 9 had deletions of only the downstream gene. We obtained different patterns of *pfhrp2*, *pfhrp3*, and their flanking gene status in samples collected from both hospitals (Table 2).

### RDT Results and *pfhrp2/pfhrp3*

All 31 samples positive for *P. falciparum* by microscopy and PCR but negative by HRP2-based RDTs had deletions of *pfhrp2* and *pfhrp3*. Conversely, all 19 *pfhrp2*-positive samples showed positive results by HRP2-based RDTs, including 12 *pfhrp3*-negative samples (Figure 2).

### Parasite Density and *pfhrp2*/*pfhrp3*

We determined the relationship between parasite density and *pfhrp2/pfhrp3* status (Figure 2). The geometric mean parasite density for the 5 *pfhrp2*-positive patients at Ghindae Hospital was 11,069 parasites/µL (95% CI 4,886–25,074 parasites/µL), which is comparable with that for the 21 *pfhrp2*-negative patients (6,111 parasites/µL, 95% CI 2,664–14,020 parasites/µL; p = 0.86). The geometric mean parasite density for the 14 *pfhrp2*-positive patients at Massawa Hospital was 2,999 parasites/µL (95% CI 1,113–8,080 parasites/µL), which was significantly lower than that for the 10 *pfhrp2*-negative patients (11,783 parasites/µL, 95% CI 4,695–29,568 parasites/µL; p = 0.021). When data for patients from the 2 hospitals were combined, geometric mean parasite densities in *pfhrp2*-positive and *pfhrp2*-negative patients were not significantly different (p = 0.085).

### HRP2 Levels and *pfhrp2/pfhrp3*

**Figure 3:**
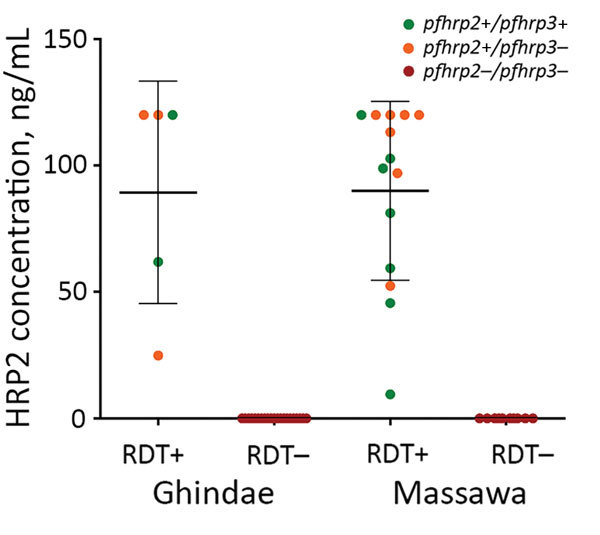
*Plasmodium falciparum* HRP2 antigen levels in relation to presence or absence of *pfhrp2/pfhrp3* genes and HRP2-based malaria RDT results, Eritrea. Horizontal lines indicate means, and error bars indicate SDs. HRP2, histidine-rich protein 2; *pfhrp, P. falciparum* histidine-rich protein; RDT, rapid diagnostic test; – negative; +, positive.

HRP2 levels, as indicated by mean MFI – bg values, were 18,885 (95% CI 16,061–21,710) for 19 *pfhrp2*-positive samples and 15.2 (95% CI 9.9–20.4) for 31 *pfhrp2*-negative samples. We found no significant difference between hospitals (p>0.05). When MFI – bg values were converted to HRP2 concentrations, *pfhrp2*-negative samples had an undetectable amount of HRP2, and *pfhrp2*-positive samples had an HRP level >9.5 ng/mL (Figure 3).

### Genetic Diversity of Parasites

**Figure 4:**
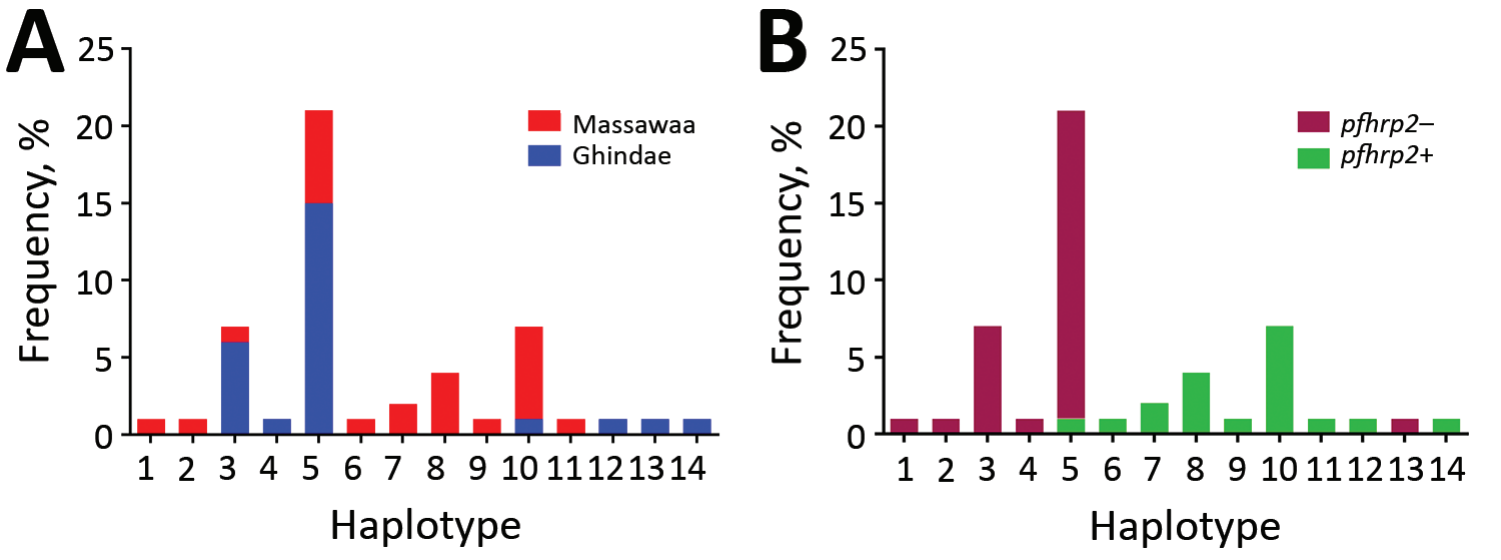
Number and frequency of *Plasmodium falciparum* haplotypes detected in patients at 2 hospitals, Eritrea, by hospital (A) and by *pfhrp2*-positive versus *pfhrp2*-negative parasite populations (B). *pfhrp, P. falciparum* histidine-rich protein; – negative; +, positive.

We determined 14 unique haplotypes for 50 samples on the basis of 7-loci microsatellite genotyping (Figure 4, panel A). All samples contained only 1 dominant haplotype. Nine haplotypes were detected for 19 *pfhrp2*-positive samples, and the maximum number of isolates sharing 1 haplotype (H10) was 7. Six haplotypes were detected for 31 *pfhrp2*-negative samples. Different haplotypes were observed for *pfhrp2*-negative and *pfhrp2*-positive populations except for haplotype H5, which was present in 20 *pfhrp2*-negative/*pfhrp3*-negative isolates and 1 *pfhrp2*-positive/*pfhrp3*-negative isolate (Figure 4, panel A). The 3 dominant haplotypes (H3, H5, and H10) were detected at both hospitals (Figure 4, panel B).

H*_E_* values were 0.44 for *pfhrp2*-positive parasite populations and 0.11 for *pfhrp2*-negative parasite populations. These values indicated an overall lower level of genetic diversity in the *pfhrp2*-negative parasite population. Three of the 7 markers in *pfhrp2*-negative parasites had H*_E_* values of 0, indicating no diversity, but only 1 marker in *pfhrp2*-positive parasites had an H*_E_* value of 0.

### Genetic Relatedness of Parasites

**Figure 5:**
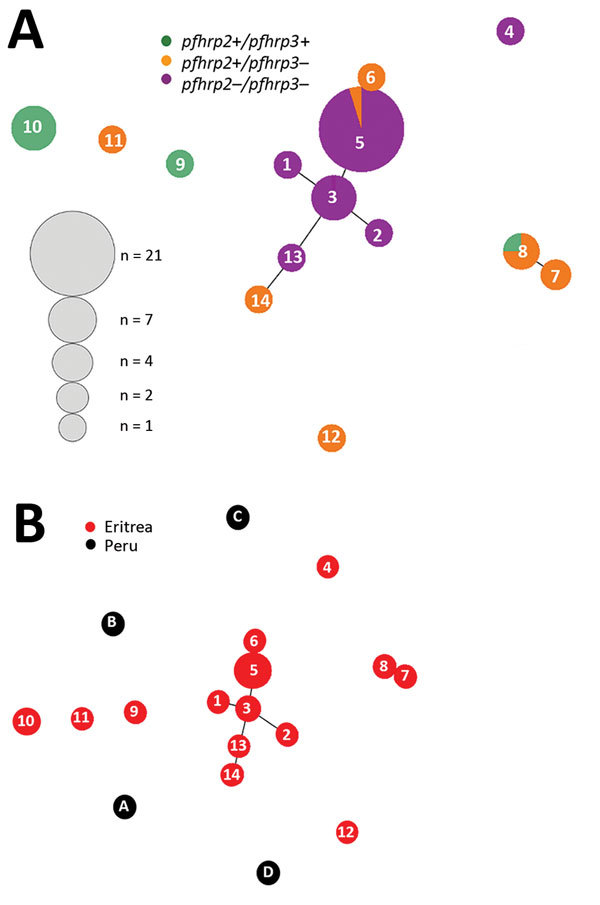
Genetic relatedness among *Plasmodium falciparum* parasite populations in Eritrea differing in *pfhrp2* and *pfhrp3* gene status (A) and comparison of parasite populations from Eritrea and Peru (B). Plots were produced by using Phyloviz software (*24*) at a cutoff value of 2 (minimum differences for 2 loci). Numbered circles indicate specific haplotypes. Circle sizes indicate number of samples with a particular haplotype. *pfhrp, P. falciparum* histidine-rich protein; – negative; +, positive.

Of 9 haplotypes for *pfhrp2*-positive parasites from Eritrea, 4 (H9, H10, H11, and H12) were scattered and 2 (H7 and H8) were genetically closely related (Figure 5, panel A). In contrast, for *pfhrp2*-negative parasites, 5 of the 6 haplotypes (H1, H2, H3, H5, and H13) were genetically closely related and formed a cluster. Also linked to this cluster were haplotypes H6 and H14, which were *pfhrp2* positive/*pfhrp3* negative*.* We found 1 unrelated *pfhrp2*-negative haplotype (H4) outside the major cluster, which indicated different genetic lineages (Figure 5, panel A). There were 2 clusters for *pfhrp3*-negative parasites.

The 14 haplotypes observed in Eritrea were unrelated to any of the 5 haplotypes observed in Peru. This finding suggested distant genetic lineages between isolates from these 2 countries (Figure 5, panel B).

## Discussion

Since malaria RDTs became available in the 1990s, growth in the number of tests, manufacturers, and volumes sold has been exponential (*25**,**26*). In parallel, the proportion of fever case-patients having access to diagnostic services before treatment has also expanded, particularly in Africa, largely attributed to the implementation of HRP2-based malaria RDTs (*3*). To continue reducing malaria transmission, use of RDTs must be expanded further, and tests must remain affordable, accurate, and user friendly.

Many factors can affect the accuracy of RDTs, and each factor should be investigated as a potential cause of false-negative results (*27*). In this instance, the Eritrea MOH had already investigated reports of false-negative results for RDTs and ruled out several possible causes (*6*). In our report, molecular and protein characterizations of prospectively collected specimens provided unambiguous evidence that confirmed that incidences of false-negative results in Eritrea were caused by a high prevalence of parasites having deletions of the *pfhrp2* and *pfhrp3* genes.

Our data showed that, in March 2016, a total of 80.8% (21/26) patients at Ghindae Hospital and 41.7% (10/24) at Massawa Hospital were infected with *pfhrp2*-negative parasites. All of these patients had undetectable levels of HRP2 in blood samples, and all showed negative results by HRP2-based RDTs and positive results by pan-pLDH–based RDTs. All samples were confirmed by microscopy and PCR as having only *P. falciparum* infections. Conversely, the remaining patient samples were *pfhrp2* positive, and all showed positive results for HRP2-based and pan-pLDH–based RDTs, and had HRP2 concentrations >9.5 ng/mL. These data confirmed that false-negative RDT results were caused by parasites lacking *pfhrp2*.

Although *pfhrp2*-negative parasites have been increasingly reported in several countries in South America, Asia, and Africa, they were mostly sporadic and showed low prevalences, except in the Amazon region of South America. Our study showed a high prevalence of parasites lacking *pfhrp2* that caused false-negative RDT results outside South America and a 100% correlation between parasites lacking the *pfhrp2* gene, the HRP2 antigen, and false-negative RDT results. These *pfhrp2* gene deletions were detected after investigations triggered by field reports of false-negative RDT results for symptomatic patients and categorically confirmed the role of this issue in malaria case management.

We showed correlations between patient demographics, parasite densities, and parasite *pfhrp2* gene status at the individual level. Our data showed that *P. falciparum* infections occurred across a range of parasite densities and age groups. All but 2 patients were symptomatic over the 2 weeks before testing, regardless of being infected with *pfhrp2*-positive or *pfhrp2*-negative parasites; the remaining 2 patients were infected with *pfhrp2*-negative parasites. Geometric mean parasite densities for *pfhrp2*-negative patients were comparable to those for *pfhrp2*-positive patients in this sample set. Further studies with larger sample sizes are required to confirm this finding.

Another useful finding of our study was the high prevalence of *pfhrp3* gene deletions in samples from Eritrea, which resulted in a high prevalence of parasites with concomitant *pfhrp2* and *pfhrp3* gene deletions. Every sample that was *pfhrp2*-negative was also *pfhrp3*-negative, which showed that 2 genes on different chromosomes were deleted in parallel. It appears that the dual-deleted parasites (*pfhrp2* negative*/pfhrp3*-negative) form clusters, and *pfhrp2*-positive/*pfhrp3*-negative parasites are scattered outside these clusters, suggesting that these parasites might have evolved from different genetic backgrounds. Although *pfhrp3* status did not affect results of RDTs used in this study, this status could affect results of other RDTs because *pfhrp3* shares sequence similarity with *pfhrp2*, and cross-reactivity between HRP2 and HRP3 has been reported for some RDTs (*28*). It is clear that regardless of the brand of HRP2-based RDTs used, parasites with dual *pfhrp2* and *pfhrp3* deletions will cause failure of RDTs because there is no possibility for cross-reactivity between HRP3 and HRP2 to occur.

A major issue is whether *pfhrp2*-negative parasites emerged locally and what is the main driving force behind their prevalence. In Peru, *pfhrp2*-negative parasites were detected in 4 of 5 major parasite populations during the late 1990s, and their prevalence increased in the absence of HRP2-based RDT pressure because microscopy was the primary diagnostic test used in that country (*23*). Spread of these parasites in Peru might have been driven by an undefined biological advantage associated with deletion of the *pfhrp2* gene.

In Eritrea, low genetic diversity of *pfhrp2*-negative parasites compared with that of *pfhrp2*-positive parasites and presence of a closely related cluster consisting of all but 1 *pfhrp2*-negative parasite suggest that clonal expansion of *pfhrp2*-negative parasites was probably caused by selection by use of HRP2-based RDTs. Because HRP2-based RDTs have been widely used in Eritrea since 2006, compliance with the recommended test before treatment is universally applied, and treatment adherence is high, conditions are ideal for selection of *pfhrp2*-negative parasites. This selection is predicted by recent mathematical modeling, which showed that exclusive use of HRP2-based RDTs exerts strong selection pressure for *pfhrp2*-negative parasites in communities and that HRP2/*P. falciparum* pan-LDH combination RDTs do not provide selection pressure for *pfhrp2*-negative parasites (*18*). Furthermore, low malaria prevalence in Eritrea might have also contributed to selection of *pfhrp2*-negative parasites once they emerge. However, historical samples were not available for a retrospective analysis of the dynamics and evolution of the *pfhrp2*-negative parasites in Eritrea.

The presence of a unique *pfhrp2*-negative parasite outside the main cluster suggests that the *pfhrp2* deletion has occurred at least twice in parasites in Eritrea. The *pfhrp2*-negative parasites in Eritrea and Peru showed distinct haplotypes, strongly suggesting de novo development of these parasites in both locations. This suggestion would imply that all malaria-endemic areas are at risk and that there is an urgent need to map the prevalence of *pfhrp2*-negative parasites to inform case management policy. The immediate mapping priority should be for countries/areas near Eritrea. It is also critical for research and development and improvements in alternative biomarkers of *P. falciparum.* Currently, there is only 1 *P. falciparum* and 1 *P. falciparum/P. vivax–*detecting RDT specific for pLDH that meet WHO procurement criteria. Most manufacturers have difficulties in producing RDTs that can consistently detect *P. falciparum* at a concentration of 200 parasites/µL by using antibodies against *P. falciparum*–pLDH (*29*).

Emergence of *pfhrp2*-negative parasites poses a major threat to malaria control programs because patients infected with these parasites are not given a correct diagnosis and treatment. Because of broad use of HRP2-based RDTs, particularly in Africa, the magnitude of selection pressure and potential scope of the problem are large. Confirming *pfhrp2/3* deletions as the cause of false-negative RDT results requires multiple PCRs performed by experienced technicians. Guidance on investigating *pfhrp2* deletions and accurate reporting is available (*21*). In addition, WHO has recently released a standard survey tool to determine whether *pfhrp2/3* gene deletions in *P. falciparum* isolated from patients with confirmed symptomatic malaria have reached a threshold triggering a change in diagnostic strategy (*30*).

In conclusion, we confirmed that frequently reported false-negative results for *P. falciparum* in specimens tested by using HRP2-based RDTs in Eritrea were caused by high proportions of parasites with deletions of *pfhrp2/pfhrp3* genes. It is clear that HRP2-based RDTs are no longer appropriate diagnostic tools for malaria case management in this country. RDTs detecting other parasite antigens, such as *P. falciparum*–pLDH, have been implemented. Our results show the need to seriously investigate reports of false-negative RDT results and conduct surveys to determine the prevalence of *pfhrp2/3* deletions in neighboring regions. International technical guidance is also needed to assist countries in planning *pfhrp2/3* surveillance activities and laboratory investigations and adapting procurement and case management strategies when *pfhrp2/3* gene deletions are confirmed.
